# Supplementary material for: Squirrel monkey responses to information from social demonstration and individual exploration using touchscreen and object choice tasks
Source: PeerJ. 2019 Nov 4;7:e7960. doi: 10.7717/peerj.7960 (PMC6836757; doi:10.7717/peerj.7960)
Supplement: Supplemental Information 1 [file peerj-07-7960-s001.docx]

**Squirrel monkey responses to information from social demonstration and individual exploration using touchscreen and object choice tasks**

**Supplemental Material**

This table illustrates the structure of a single research session for both experiments.

**Supplemental Table S1. Structure of a single research session for Experiments 1 and 2**

| Problem number | Trial number | In the Social condition, performed by | | In the Individual condition, performed by |
| --- | --- | --- | --- | --- |
| 1 | T1 (information trial) | Human | Puppet | Subject |
|  | T2 | Subject | | Subject |
|  | T3 | Subject | | Subject |
|  | T4 | Subject | | Subject |
|  | T5 | Subject | | Subject |
| 2 | T1 (information trial) | Human | Puppet | Subject |
|  | T2 | Subject | | Subject |
|  | T3 | Subject | | Subject |
|  | T4 | Subject | | Subject |
|  | T5 | Subject | | Subject |
| 3 | T1 (information trial) | Human | Puppet | Subject |
|  | T2 | Subject | | Subject |
|  | T3 | Subject | | Subject |
|  | T4 | Subject | | Subject |
|  | T5 | Subject | | Subject |
| 4 | T1 (information trial) | Human | Puppet | Subject |
|  | T2 | Subject | | Subject |
|  | T3 | Subject | | Subject |
|  | T4 | Subject | | Subject |
|  | T5 | Subject | | Subject |

**Experiment 1: Touchscreen**

*Post hoc analysis: interaction between information type and source (success measure)*

The main model with success as the response variable found an interaction between information type and source (human or puppet demonstration, and individual condition; Helmert contrasts). Shown in Supplemental Table S2 are the results of a post hoc analysis using *emmeans*. Results are given on the log odds ratio scale.

**Supplemental Table S2. Pairwise contrasts for win vs lose information trials by source in the touchscreen task (T2 success measure)**

| **Information type** | **Contrast** | **Estimate** | **SE** | **Z ratio** | ***p* value** |
| --- | --- | --- | --- | --- | --- |
| Win | Puppet vs human | −0.36 | 0.18 | −2.0 | 0.045* |
|  | Individual vs (puppet + human) | 0.42 | 0.30 | 1.4 | 0.17 |
| Lose | Puppet vs human | −0.14 | 0.17 | −0.84 | 0.40 |
|  | Individual vs (puppet + human) | −0.66 | 0.29 | −2.3 | 0.021* |

**Experiment 2: Three-dimensional objects**

We pre-registered our analyses for Experiment 2, and reported the results in the paper. We also built additional models that incorporated a fuller suite of random effects, some of which were not pre-registered. Here we present the results of these analyses.

*Post hoc analysis: interaction between information type and source (WSLS measure)*

To explore the interaction from the above pre-registered model examining the effects of information type and source, we carried out post hoc tests using *emmeans*. The results are shown below in Supplemental Table S3. Results are given on the log odds ratio scale.

**Supplemental Table S3. Pairwise contrasts for the interaction between information type and source in the 3D object task**

| **Source** | **Contrast** | **Estimate** | **SE** | **Z ratio** | ***p* value** |
| --- | --- | --- | --- | --- | --- |
| Individual | Lose – win | −2.2 | 0.32 | −6.8 | <0.0001* |
| Social | Lose – win | 0.034 | 0.22 | 0.16 | 0.998 |

**Experiments 1 and 2: Combined data set**

*Post hoc analysis: interaction between information type, source, and presentation medium in combined 2D-3D data (WSLS measure)*

In the main manuscript, we reported the results of a GLM that examined the effects of information type, source, and presentation medium (and their interactions). To clarify the three-way interaction between information type, source, and presentation medium, we carried out post hoc tests using *emmeans*. The three-way interaction indicates that the source-information type interactions differed in the two presentation mediums. This is supported by the results of the separate analyses of the WSLS variable for Experiments 1 and 2: although significant source-information type interactions were identified in both experiments (due to a larger effect of better performance following win trials, compared with lose trials, in the individual, compared with the social, condition), this effect was stronger in Experiment 2. The results are shown below in Supplemental Table S4. Results are given on the log odds ratio scale.

**Supplemental Table S4. Pairwise contrasts for the interaction between information type, source, and presentation medium in the combined 2D-3D data (WSLS measure)**

| **Presentation medium** | **Source** | **Information type** | **Estimate** | **SE** | **Z ratio** | ***p* value** |
| --- | --- | --- | --- | --- | --- | --- |
| Tablet (2D) | Individual | Lose – win | −1.2 | 0.12 | −10.1 | <0.0001* |
|  | Social | Lose – win | −0.71 | 0.12 | −5.7 | <0.0001* |
| Cups (3D) | Individual | Lose – win | −2.1 | 0.32 | −6.7 | <0.0001* |
|  | Social | Lose – win | 0.020 | 0.22 | 0.09 | 1.00 |

*Post hoc analysis: interaction between source and presentation medium in combined 2D-3D data (repeats measure)*

In the main manuscript, we reported the results of a GLMM that examined the effects of information type, source, and presentation medium (and their interactions) on monkeys’ tendency to repeat. To clarify the only significant interaction from that model, an interaction between source and presentation medium, we carried out a post hoc analysis using *emmeans*, results of which are shown in Supplemental Table S5. This indicates that monkeys’ greater likelihood of repeating their own actions, compared with repeating those of a demonstrator, was stronger for the 3D object choice task than the 2D touchscreen task, and is supported by the separate analyses from Experiments 1 and 2. Results are given on the log odds ratio scale.

**Supplemental Table S5. Pairwise contrasts for the interaction between source and presentation medium in the combined 2D-3D data (repeats measure)**

| **Presentation medium** | **Source** | **Estimate** | **SE** | **Z ratio** | ***p* value** |
| --- | --- | --- | --- | --- | --- |
| Tablet (2D) | Social – individual | −0.23 | 0.17 | −1.4 | 0.44 |
| Cups (3D) | Social – individual | −1.1 | 0.24 | −4.6 | <0.0001* |
